# Practices, beliefs, and attitudes of clinicians in prescribing direct oral anticoagulants for obese adults with atrial fibrillation: a qualitative study

**DOI:** 10.1007/s11096-023-01583-z

**Published:** 2023-05-30

**Authors:** Fahad Shaikh, Rochelle Wynne, Ronald L. Castelino, Sally C. Inglis, Patricia M. Davidson, Caleb Ferguson

**Affiliations:** 1 School of Nursing, Faculty of Science, Medicine & Health, University of Wollongong, Wollongong, NSW Australia; 2 The Royal Melbourne Hospital, Parkville, VIC Australia; 3 Faculty of Medicine and Health, University of Sydney, Sydney, NSW Australia; 4 Pharmacy Department, Blacktown Hospital, Western Sydney Local Health District, Blacktown, NSW Australia; 5 Improving Palliative, Aged and Chronic Care Through Clinical Research and Translation (IMPACCT), University of Technology Sydney, Sydney, NSW Australia; 6 University of Wollongong, Wollongong, NSW Australia

**Keywords:** Anticoagulant, Atrial fibrillation, Direct oral anticoagulants, Obesity, Qualitative, Research

## Abstract

**Background:**

Atrial fibrillation (AF) and obesity affect over 60 and 650 million people, respectively.

**Aim:**

This study aimed to explore clinician practices, beliefs, and attitudes towards the use of direct oral anticoagulants (DOACs) in obese adults (BMI ≥ 30 kg/m^2^) with AF.

**Method:**

Semi-structured interviews via video conference were conducted with multidisciplinary clinicians from across Australia, with expertise in DOAC use in adults with AF. Clinicians were invited to participate using purposive and snowball sampling techniques. Data were analysed in NVIVO using thematic analysis.

**Results:**

Fifteen clinicians including cardiologists (n = 5), hospital and academic pharmacists (n = 5), general practitioners (n = 2), a haematologist, a neurologist and a clinical pharmacologist participated. Interviews were on average 31 ± 9 min. Key themes identified were: *Health system factors in decision-making* Disparities between rural and metropolitan geographic areas, availability of health services, and time limitations for in-patient decision-making, were described; *Condition-related factors in decision-making* Clinicians questioned the significance of obesity as part of decision-making due to the practical limitations of dose modification, and the rarity of the extremely obese cohort; *Decision-making in the context of uncertainty* Clinicians reported limited availability, reliability and awareness of primary evidence including limited guidance from clinical guidelines for DOAC use in obesity.

**Conclusion:**

This study highlights the complexity of decision-making for clinicians, due to the limited availability, reliability and awareness of evidence, the intrinsic complexity of the obese cohort and limited guidance from clinical guidelines. This highlights the urgent need for contemporary research to improve the quality of evidence to guide informed shared decision-making.

**Supplementary Information:**

The online version contains supplementary material available at 10.1007/s11096-023-01583-z.

## Impact statements

- Beliefs and attitudes of a clinician may affect their decision-making process.
- Clinicians are faced with dealing with the intrinsic complexities of obesity with limited guidance provided by clinical guidelines.
- The increasing rate of individuals with concomitant obesity and AF highlights the urgency and need for improvement in the quality and availability of evidence to help guide clinicians in their decision-making.

## Introduction

Atrial fibrillation (AF) and obesity are two of the most prevalent conditions in the world, affecting 60 and 650 million people globally, respectively [1, 2]. It is estimated that obesity increases the risk of developing AF by up to 50% and almost one in five cases of AF are attributed to obesity, to the extent that there is a 4–5% increase in AF risk for each incremental increase in body mass index (BMI) [3–6].

Obesity is associated with an increased rate of unplanned, cardiovascular-related, and all-cause hospital admissions in patients with AF [7]. Studies highlight the complex interplay of pathophysiological mechanisms with changes in hemodynamic, autonomic, and inflammatory responses and structural remodelling [8–14] influencing the pharmacokinetics of medications used in AF management [15].

Direct oral anticoagulants (DOACs) have been preferred over warfarin by clinical guidelines, due to their superior safety profile. They also provide practical advantages such as no need for prothrombin time (PT) or international normalized ratio (INR) monitoring, and fewer drug interactions, thus resulting in an increasing utilisation in clinical practice [16]. However, despite these advantages, there is still an alarming rate of prescribing errors, where almost 1 in 5 patients on DOACs have experienced a prescribing error such as under or overdosing, duplication of therapy and contraindications.

Furthermore, there is disagreement in pharmacokinetic reports of DOAC peak and trough plasma concentrations when comparing normal and obese adults [17–20]. This disagreement pervades opinion regarding the clinical effect of obesity in the context of DOACs, given the lack of long-term outcome data and variability in prescribing [21, 22]. Clinician perceptions of uncertainty, the need for individualised decision-making, and attitudes toward delegation of responsibility may be factors in reluctance to treat with anticoagulants [23].

Clinicians formulate distinct intellectual responses to available scientific evidence, the prospect of emerging evidence, monitoring mechanisms and safety concerns, especially concerning the potential to cause harm and accountability for that harm [24]. The inherent bias in the context of no data to support viewpoints influences racial and socioeconomic prejudice and prescribing [24, 25]. Although views on decision-making and prescribing anticoagulants in patients with AF have been explored in several studies [23, 24, 26–34], there is very limited research investigating clinicians’ perspectives on the effect of obesity on anticoagulants in patients with AF.

### Aim

This study aimed to explore clinician practices, beliefs, and attitudes towards the use of direct oral anticoagulants (DOACs) in obese adults (BMI ≥ 30 kg/m^2^) with AF.

### Ethics approval

The study was approved by Western Sydney Local Health District (REF: 2020/ETH03065; 06/01/2021) and Western Sydney University (REF: RH14416; 12/07/2021) Human Research Ethics Committee.

## Method

### Design

One-to-one semi-structured interviews with open-ended questions were conducted to explore multidisciplinary clinicians’ perceptions (Supplemental Table 1: Interview Guide). This study follows the standards for reporting qualitative research (SRQR) [35].

### Setting & sample

Clinicians from across Australia with expertise in the use of DOACs in patients with AF, from either a medical, pharmacy or nursing background, were invited to participate via a combination of purposive and snowball sampling techniques.

### Recruitment procedure

An initial list of clinicians that the researchers knew who had expertise on the topic, through their role in expert peak bodies and guideline development at a national and international level, were identified. Invitations for an interview sent via email included an outline of the proposed research. Clinicians who agreed were asked to sign and return the consent form, and a mutual time was confirmed to conduct one-on-one interviews via video conference. After the interview, clinicians were asked to recommend other clinicians for an interview, who had expertise on the topic. Interviews were recorded, and hand-written field notes were taken to supplement verbatim interview transcripts as interviews progressed. Clinicians could withdraw from the study at any point.

### Data analysis

Concurrent interviews and data analysis enabled efficient identification of the point of data saturation where no new themes emerged from ongoing interviews. Verbatim transcriptions were checked for validity against the audio and manually recorded data. Transcriptions were uploaded and managed using NVIVO software (QSR International, 2020). Thematic analysis was conducted as per the Braun and Clarke (2015) framework [36]. Each transcript was read and coded independently by two authors (FS, CF) to identify initial themes subsequently confirmed by a third member of the team (RW).

## Results

A total of 16 clinicians; cardiologists (n = 5), hospital and academic pharmacists (n = 5), general practitioners (n = 2), a haematologist, a neurologist, a clinical pharmacologist, and a clinical nurse consultant (CNC), agreed to participate but the CNC withdrew on the premise of a lack of experience focused specifically on the intersection of obesity and AF. Nine (60%) were male and experience ranged from relatively junior (3 years of practice) to senior clinicians working in their specialty for up to 39 years (median: 22 years). Apart from one, all the clinicians were based in a metropolitan city; Sydney (53%), Melbourne (20%), Adelaide (13%) and Hobart (7%). Interviews lasted for 31 ± 9 min, and three key themes were identified: 1) health system factors in decision-making, 2) condition-related factors in decision-making, and 3) decision-making in the context of uncertainty, discussed in detail below.

### Health system factors in decision making

Clinicians in rural general practice settings highlighted disparities within the healthcare system as barriers to decision-making. These included access and availability of health services and longer waiting times for specialist services in rural/regional areas when compared to metropolitan areas (i.e. Sydney or Melbourne), influencing delays in the initiation of anticoagulation.

“*People who are right in the middle of Melbourne and Sydney, they just tend to refer a lot of people. And of course, you can’t afford to be like that in a region like ours, or if you’re more rurally based. I mean, you’d be waiting quite a long time to get access and care for the patients*”- General Practitioner 01.

Clinicians from hospital settings questioned the need for urgency in treatment decisions when prescribing DOACs in the context of obesity. Some acknowledged the importance of considering obesity when decision-making, but their primary concern was related to the significance of incorporating obesity into decision-making during time-constrained, acute hospitalisations. Time was a barrier to effective decision-making and clinicians reported frequently having to overlook potentially influential factors because of the inability to obtain immediate results.

“… *Obesity is important, and everyone will acknowledge that, but at the end of the day, they’re managing what they have in front of them that is distressing symptomatically and clinically to the patient, and it’s impacting on them functionally, and that they’re easier things to deal with because you can initiate therapy for that. But dealing with obesity, that’s a whole other thing, and that can’t be addressed, by any practical means, like there’s nothing that you can practically do in hospital admission, that is going to have a significant impact on that patient’s obesity status. that’s still a long process… you’re limited to what you can do*” – Pharmacist 01.

### Influence of obesity on decision-making

Clinicians emphasised the rarity of the obese AF patient population as a factor that leads them to question the clinical significance of obesity as part of their decision-making. Clinicians often referred to obese patients as a “*rare cohort”*, with most expressing doubts related to dealing with obese AF patients.

*“I don’t know very many patients with AF who are in the morbidly obese category*”- General Practitioner 02.

“*… they are a rarer group, even though obesity is on the rise, but the morbidly obese patient is, a relatively smaller part of the population*” – Pharmacist 01.

Some clinicians acknowledged the importance of taking into consideration the associated complexities of care for obese adults. This was generally discussed in terms of the multiple co-morbidities that exist in obese adults, often referred to as a “*special population*” or “*cohort own on their own*”. However, this also led to some clinicians questioning the importance of obesity in the overall management strategy, secondary to limitations on the practical implementation of dose adjustments and services provided.

“*I wouldn’t worry about it. So, I worry much more about the renal side of things because the renal side of things can change so quickly… But when I’m thinking about body weight in the dosing of a DOAC, I think about it at one point in time, which is at the time when the initial prescription is made. At that point, I’ll consult and then pretty well, after that, I’ll just forget about it.*”- Clinical Pharmacologist.

### Decision-making in the context of uncertainty

Participants were concerned about the availability, reliability, and high level of uncertainty of evidence for DOAC use in obesity. They questioned the quality of obesity evidence due to limitations in clinical trial inclusion criteria, conflicting evidence, and variability in advice. The lack of direction from guidelines because of the unknowns in this aspect of practice was noted, often disliking the approaches taken as they felt ‘left in the dark’ and generally on their own to make decisions.

“*Yeah, I’m aware of that as a guideline, and I really don’t like it because it’s not practical. There are a lot of patients around that weight, and they’re at really high risk, …. And we’re saying, Ah, sorry, we just haven’t done the studies. And so, you’ll have to go on warfarin. And then there are the issues of the blood test. So, I actually think it’s quite discriminatory, that we haven’t addressed this more seriously than, other than saying, ah, we don’t have the data, we do it. And the fact that we haven’t done it, I think is wrong*” – Clinical Pharmacologist.

Clinicians stated the presence of a lag period from evidence generation to implementation or awareness of the evidence in practice, impacted decision-making.

“*… guidelines are always a tricky thing and I always say, just remember the guidelines are just a guide and, almost as soon as they’re written, they’re out of date*”- Pharmacist 01.

Treatment outcome appeared to be a primary concern for clinicians and the main factor taken into consideration when decision-making. Possibilities included the risk–benefit of anticoagulation, the clinical significance of obesity influencing DOAC effectiveness and known DOAC safety profiles. Concerns about bleeding risk with dose modification led to decision-making based on the first principal approach. This would vary depending on individual clinician beliefs regarding the quality of evidence, combined with past clinical and prescribing experience.

“*Clinicians that have had a patient experience a negative effect will employ a more cautious approach, as opposed to some who hasn’t experienced this*” – Pharmacist 01.

Whilst acknowledging the lack of evidence/data and limitations in predicting future outcomes, some clinicians believed that the “*absence of data, is not the absence of effect”*.

“*We don’t have any randomized control data for DOACS against placebo or nothing. We do have a limited number of things that were, summarized, in a meta-analysis and on which basis the recommendations for giving, oral anticoagulation for non-valvular AF have been made. In those studies, which are much smaller than the DOAC studies in general, as far as I’m aware, there was no mention or sort of segregation of people who were morbidly obese. So, in other words, we don’t have data on morbidly obese people with Warfarin, but at least with Warfarin you are dealing with, trying to get people to a certain INR, not sort of give them a dose. I do not subscribe to the fact that the absence of data means the absence of effect*”—Cardiologist 01.

Clinicians often stressed the importance of an integrated, multidisciplinary approach and shared decision-making to provide the optimal level of care for the patient. This included collaborating and relying on interprofessional expertise, acknowledging their respective professional scope of practice and ensuring continuity of care. Whilst accounting for preferences and past experiences of patients, considering current evidence and risk–benefit of being on a different agent, most often patients would trust the preference of the prescribing doctor.

“*Most of the patients I see generally, I explain the process that we are going through. They’re generally pretty comfortable with the decisions I make. I used to give them a whole lot of information about the various options. And, in the end, they generally went with what I recommended anyway.*” – Cardiologist 02.

“*Look… I’d probably consult, the ward pharmacist at a minimum. Get them to do that hard work and look up the dosing changes or which agent we should use, one preferred to the other. Ultimately I sort of make a decision based on what evidence they’ve shown*” – Neurologist.

“*So, I actually have a chat with a pharmacist, because they’re always really good with giving advice. Then my understanding is that you have warfarin’s, like at the extremes of weight…. But warfarin is really difficult to, administer if you can’t test for it. And often, if someone’s morbidly obese, you can’t… Getting blood out of them is incredibly difficult. So, I would usually, under those circumstances, consult either any regulation specialist or hematology, that would be my threshold of saying, I really much prefer to use DOAC*” – Clinical Pharmacologist.

## Discussion

### Statement of key findings

This exploration of the practices, beliefs, and attitudes of clinicians concerning the use of DOACs in obese adults with AF highlights the spectrum of views about considering obesity as part of the clinician’s decision-making process. While almost all clinicians acknowledged the negative impacts of obesity on AF, only a few considered obesity as a key component in their decision-making for several reasons. Challenges within the healthcare system, the complexity of patients presenting conditions, the perceived rarity of the obese-AF population and the fact that decision-making takes place in the context of uncertainty were amplified by time limitations and geographical discrepancies in access to specialist healthcare advice and services.

### Interpretation

In acute care facilities where demand is high, the urgency of cases clinicians need to treat perpetually limits time. Referral to outpatient services (e.g., anticoagulation clinics, home medicine review), and follow-up appointments in primary care to ensure appropriate and adequate treatment are proposed solutions [37–39] but these findings support the notion that geographical access challenges in Australia are a longstanding issue, evidenced by poorer health outcomes in rural versus metropolitan regions [40]. Specialist referral rates are significantly lower in “very remote” compared to “major city” (22 versus 143 per 100,000 population) areas. Complicating this is the higher prevalence of obese adults in rural regions (70%) compared to “major cities” (65%) which burdens clinicians in rural settings with limited resources [40]. Telehealth in rural areas improves options via timely advice for decision-making. Several studies demonstrated successful implementation, continuity of care, and improved patient outcomes and satisfaction, with telehealth programs for anticoagulation care during COVID-19 [41–44].

Coupled with the health system factors, the clinician’s perception of the prevalence of the obese-AF population also greatly influenced the clinician’s view on the clinical significance of the effect of DOACs in the obese-AF population. However, this unexpected perception would oppose the current data from international registries and large population studies such as the ARIC, EORP-AF, CardioCHUVI-AF and Gulf SAFE registries which show that up to 46% of the AF population were obese [8, 45]. Furthermore, the well-known negative impact and risk associated with AF, as the clinicians acknowledged, coupled with the increasing prevalence of AF and obesity in Australia (5 and 31.3% respectively), would suggest that the obese-AF population would be similar to the above-mentioned prevalence [46, 47]. These findings highlight the gap in the current literature on the epidemiology of obese-AF adults in countries where both obesity and AF are on the rise, such as Australia.

Decision-making in the context of uncertain and often conflicting evidence was a major theme highlighted by participants due to either a lack of awareness of the latest evidence, personal conviction regarding the quality and reliability of data from trials and other studies, non-specificity in guidelines, and recognition that even the most contemporary guidelines are rapidly outdated as evidenced by the translational lag between evidence generation and implementation [48].

This is further compounded by the conflicting findings in published systematic reviews on this topic [22, 49], and the unequal distribution of the weight classes in clinical trials such as ARISTOTLE, RELY, and ROCKET-AF [50–52]. In fact, the majority of patients enrolled in the RELY-AF trial (up to 80%), were between 50-100 kg [53]. Patients that were > 140 kg were under-represented as only 1.4% of the sample in the ARISTOTLE trial.

As such, both the International Society on Thrombosis and Haemostasis (ISTH) and the European Society of Cardiology (ESC) Working Group on Thrombosis have questioned the use of DOACs in extremely obese adults (i.e. BMI ≥ 40 kg/m^2^), due to the extremely limited or absent clinical data [54]. The ISTH has suggested that DOAC should not be used in a BMI of > 40 kg/m^2^ or > 120 kg due to the limited clinical data available [55].

The introduction of “living guidelines” provides an option for timely, contemporary evidence for clinicians [56, 57] that could negate translational lag. The importance and benefit of interprofessional collaboration and shared expertise in decision-making were perceived to optimise patient care in this study. Dreijer et al., tested a multidisciplinary antithrombotic team that significantly improved adherence to anticoagulant guidelines amongst prescribing clinicians [58]. Shared decision-making allows patients to take part in the process by considering their values and preferences [59]. This ultimately improves patient satisfaction, trust and adherence to medications [60]. Complimentary mechanisms augment patient care, improving health outcomes [61].

### Strengths and weaknesses

The practices, beliefs, and attitudes of clinicians are under-investigated. The recruitment of diverse clinicians for standardised interviews provided rich substantive information to improve our understanding of key concerns for managing obese adults with AF. Although the inherent limitation of a qualitative study is the limited generalisability of these themes uniformly in all countries. However, the themes generated in this study have the potential for transferability to future studies conducted in countries that have similar patient demographics to Australia and may also provide clinician-instigated hypotheses, thus generating ideas for future research.

### Further research

Consistent with previous research, evidence uncertainty, shared decision-making, personal preferences, and safety concerns are not new issues for prescribing clinicians [23, 24, 30, 32–34]. This research reveals the additional level of complexity obesity adds to decision-making. Whether it is safe and efficacious, to initiate or change dosing and choice of anticoagulant according to weight is unknown and influenced by internal and external barriers that prevent obesity from being considered in decision-making. The absence of definitive guidelines substantiates the need for timely robust research examining the effect of obesity on DOACs in the context of AF. Stratification in trials is an immediate remedy to ensure comparable numbers of participants from BMI categories.

## Conclusion

A range of views and perceptions of barriers to the incorporation of obesity as part of the decision-making process in AF were identified in this study. Findings highlight the complexity of decision-making for clinicians, due to limitations in evidence, the intrinsic complexity of the obese cohort and the absence of robust practice guidelines. To generate more conclusive evidence on the use of DOACs in the context of obesity, future research must focus on testing effects according to categories of BMI and “living” guidelines must inform shared decision-making, crucial to stemming the negative outcomes associated with endemic obesity and AF.

## Supplementary Information

Below is the link to the electronic supplementary material.

Supplementary file1 (DOCX 21 KB)
