# Supplementary material for: Practices, beliefs, and attitudes of clinicians in prescribing direct oral anticoagulants for obese adults with atrial fibrillation: a qualitative study
Source: Int J Clin Pharm. 2023 May 30;45(4):962–9. doi: 10.1007/s11096-023-01583-z (PMC10228882; doi:10.1007/s11096-023-01583-z)
Supplement: Supplementary file 1 — Supplementary file1 (DOCX 21 KB) [file 11096_2023_1583_MOESM1_ESM.docx]

Supplementary Material

# Table 1: Interview Guide

| **Clinician Type** | **Questions** |
| --- | --- |
| **Prescribers** | 1. Could you give some background information about yourself, including how many years have been practicing in your specialty and in particular your experience with managing patients with atrial fibrillation? 2. What are some of the key factors that influence your decision making and prescribing practices with patients with AF? 3. Can you explain your clinical approach towards your overweight and/or obese patients with AF that you deal with on a regular basis? 4. Can you explain your pharmacological preference for different weight classes with respect to anticoagulants? 5. What are your views on obesity and their effect on NOACs? 6. How significant do you think the effect body weight has on outcome in patients prescribed with NOACs? 7. Have you had any patient complain of an adverse drug reaction? And do you think their weight played a role in this? 8. Could you please share your clinical approach for any patient that has had a recent hospitalization and/or adverse effect due to their AF? 9. Are you aware of any guidelines or literature which suggests body weight may or may not influence the outcome of patients with AF? If so, what do they suggest and if you agree with it? |
| Cardiologist  Nurse Practitioner  Haematologist  Neurologist  General Practitioner |  |
| **Non-Prescribers** | 1. Could you give some background information about yourself, including how many years have been practicing in your specialty and in particular your experience with managing patients with atrial fibrillation? 2. Can you explain your clinical approach towards your overweight and/or obese patients with AF that you deal with on a regular basis? 3. Can you explain your pharmacological preference for different weight classes with respect to anticoagulants? 4. What are your views on obesity and their effect on NOACs? 5. How significant do you think the effect body weight has on outcome in patients prescribed with NOACs? 6. Have you had any patient complain of an adverse drug reaction? And do you think their weight played a role in this? 7. Could you please share your clinical approach for any patient that has had a recent hospitalization and/or adverse effect due to their AF? 8. Are you aware of any guidelines or literature which suggests body weight may or may not influence the outcome of patients with AF? If so, what do they suggest and if you agree with it? |
| Pharmacist  Clinical Nurse Specialist |  |

# Table 2: Full interview quotes

| ***Health system factors in decision making*** | |
| --- | --- |
| General Practitioner 01 | “*Because I do get the impression from people who are right in the middle of Melbourne and Sydney, that they just tend to refer a lot of people. And of course, you can't afford to be like that in a region like ours, or if you're more rurally based. I mean, you'd be waiting quite a long time to get access and care for the patients*”- |
| Pharmacist 01 | “*So, you know, you will say that there will be referrals, for patients to address that, but even still, obesity clinics and services and the clinicians that are specialised in this space, are still far and few between and the waiting lists to get into those places are I'm told are quite long*”  “. . . *obesity is important, and everyone will acknowledge that, but at the end of the day, they're managing what they have in front of them that is distressing symptomatically and clinically to the patient, and it's impacting on them functionally, and that they're easier things to deal with because you can initiate therapy for that. You know, pharmacologically, non-pharmacologically. But dealing with the obesity, that's a whole other thing, and that can't be addressed, to any practical means, like there's nothing that you can practically do in an, hospital admission, that is going to have a significant impact on that patient's obesity status. You know, that, that's still a long process-...relative to what you can do fairly quickly. So, it, it's important clinically, but in terms of management in a hospital where you have, , a relatively short period of time, you don't have months, to kind of reverse obesity in somebody that's really suffering. Yeah, you're limited to what you can do*” |
| ***Influence of obesity in decision-making*** | |
| Cardiologist 01 | *“I have not, in my practice, changed the dosage of those drugs for morbid obesity, but I don't treat anyone who's obese, so it hasn't actually come to in my own practice”* |
| General Practitioner 02 | *“I don't know very many patients with AF who are the morbidly obese category”* |
| Pharmacist 01 | “. . . *they are a rarer group, even though obesity is on the rise, but the morbidly obese patient is, you know, a relatively smaller part of the population”* |
| Clinical Pharmacologist | “*I wouldn't worry about it. So, I worry much more about the renal side of things because the renal side of things can change so quickly….. There are so many more things that would worry me about the kidney function that could then impact upon the DOAC dosing, that that is the very much front and centre of my mind. But when I'm thinking about body weight in the dosing of a DOAC, I think about it at one point of time, which is at the time when the initial prescription is made. At that point, I'll consult and then pretty well, after that, I'll just forget about it.”* |
| ***Decision-making in the context of uncertainty*** | |
| Clinical Pharmacologist | *“Yeah, I'm aware of that as a guideline, and I really don't like it because it's not practical. That's where I think we need to address this, because 120 kilos is pretty easy to say. And there are a lot of patients around at that weight, and they're at really high risk, because they've got spaces, because they're, they don't move around very much. They have, lots of, of tissue pushing up against the blood vessels. They've got lots of injuries, they've got lots of medications on-board. There's lots of reasons for them to then have issues with thrombosis. And we're saying, "Ah, sorry, we just haven't done the studies." And so, you'll have to go on warfarin. And then there's the issues of the blood test. So, I actually think it's quite discriminatory, that we haven't addressed this more seriously than, other than saying, ah, we don't have the data, we do it. So, I think either we go and get the data by just doing trials, or we get the data in other ways. So, there are, there are ways, epidemiological ways to look at safety. It's not that difficult to do. And the fact that we haven't done it, I think is wrong”* |
| Pharmacist 01 | *“. . . the guidelines are always a tricky thing and I always say, you know, just remember the guidelines are just a guide and, almost as soon as they're written, they're out of date”*  “*Clinicians that have had a patient experience a negative effect will employ a more cautious approach, as opposed to some who hasn’t experienced this”* |
| Cardiologist 01 | “*I was saying that we don't have any randomized control data for DOACS against placebo or nothing. Okay? We do have a limited number of things that were, summarized by, in a meta-analysis and on which basis the recommendations for giving, oral anti-coagulation for non-valvular AF have been made. In those studies, which are much smaller than the DOAC studies in general, as far as I'm aware, there was no mention or sort of segregation of people who were morbidly obese. So, in other words, we don't have data on morbidly obese people with Warfarin, but at least with Warfarin you are dealing with, trying to get people to a certain INR, not sort of give them a dose. With DOACs, we're not using a dose. I do not, I do not subscribe to the fact that the absence of data means the absence of effect, and in the absence of data, I have just used that and tried to reduce obesity as, as a matter of fact. So that's my management.”* |
| Cardiologist 02 | “*The... most of the patients I see... generally , I explain what you know, the process that we are going through. They're, they're generally pretty comfortable with the decisions I make. I used to give them a whole lot of information about the various options. And, in the end they generally went with what I recommended anyway.”* |
| Neurologist | “*look I'd probably, if given ... these patients mostly in hospital, I'd probably consult, the ward pharmacist at a minimum. Get them to do that hard work and look up the dosing changes or which agent we should use, one preferred to the other. Ultimately I sort of make a decision based on what evidence they've shown*” |
| Clinical Pharmacologist | “*so, the way I would approach it is, so I actually have a chat with a pharmacist, because they're always really good with giving advice. But I understand there's a busy, you can use rivaroxaban up to a certain weight, and I would choose to go down that line, if I could. And then my understanding is that you have warfarin’s, like at the extremes of weight, warfarin is recommended. But warfarin is really difficult to, administer, if you can't test for it. And often, if someone's morbidly obese, you can't... Getting blood out of them is incredibly difficult. And so, I would usually under those circumstances, consult either any regulation specialist or hematology, that would be my threshold of saying, I really much prefer to use DOAC*” |
